# Analyzing Trends in Suicide Attempts Among the Pediatric Population in the United States: A Study Using CDC’s Youth Risk Behavior Surveillance System (YRBSS) Database

**DOI:** 10.7759/cureus.44099

**Published:** 2023-08-25

**Authors:** Abimbola E Arisoyin, Ayomide H Adeyemi, Okelue E Okobi, Adeyemi H Alaga, Oluwabukola J Adekunle, Olamide O Ajayi, Funmi Aladeniyi, Esther O Oni, Emeka Okobi, Matayebi Okeaya-Inneh, Claire-Louise Young

**Affiliations:** 1 Internal Medicine, College of Medicine, University of Lagos, Lagos, NGA; 2 Medicine, VN Karazin Kharkiv National University, Kharkov, UKR; 3 Family Medicine, Larkin Community Hospital Palm Springs Campus, Miami, USA; 4 Family Medicine, Medficient Health Systems, Laurel, USA; 5 Family Medicine, Lakeside Medical Center, Belle Glade, USA; 6 Psychiatry, Grand River Hospital, Kitchener, CAN; 7 General Practice, College of Medicine, University of Lagos, Lagos, NGA; 8 Internal Medicine, Obafemi Awolowo College of Health Sciences, Olabisi Onabanjo University, Sagamu, NGA; 9 Department of Medicine, American University of Antigua, Antigua, ATG; 10 General Practice, Ladoke Akintola University of Technology, Ogbomoso, NGA; 11 Dentistry, Ahmadu Bello University Teaching Hospital, Abuja, NGA; 12 Family Medicine, Garki Hospital Abuja, Abuja, NGA; 13 Family Medicine, Hamilton Medical Center, Dalton, USA

**Keywords:** trends analysis, cdc's yrbss database, united states, pediatric, suicide attempts

## Abstract

Background

Suicide is a significant public health concern among the pediatric population in the United States. This study aims to comprehensively analyze suicide attempts among adolescents using data from the Youth Risk Behavior Surveillance System (YRBSS) maintained by the Centers for Disease Control and Prevention (CDC).

Methods

The pediatric population of grades 9-12 students (13-17 years old) was included in the study population, and data were collected from multiple cycles of the YRBSS survey. Descriptive statistics and time-trend analyses were conducted to examine attempted suicide rates based on location, gender, race/ethnicity, school grade level, and sexual orientation.

Results

Significant variations in attempted suicide rates were observed among different demographic groups. In 2021, of the subset with suicide attempt, females reported a higher prevalence of attempted suicide (13.3%, n=211), while males exhibited a lower rate (6.6%, n=104). Of the total studied population in 2021, Palau had the highest attempted suicide rate (25.2%, n=3924), followed by the Northern Mariana Islands (17.6%, n=2740). Over 1991-2021, no significant location-based variations were observed. In 2021, American Indian/Alaska Native adolescents had the highest attempted suicide rate at 16% (n=2491), followed by Black adolescents (14.5%, n=2258). Ninth-grade students reported higher rates in 2021 (11.6%, n=1806). Adolescents reporting both opposite-sex (36.7%, n=5715) and same-sex-only sexual contacts or both (32.9%, n=5123) exhibited notably higher rates in 2021.

Conclusion

This study highlights alarming attempted suicide rates in the US pediatric population, emphasizing the need for tailored prevention efforts and mental health support. It offers essential guidance for policymakers, researchers, and mental health professionals in developing evidence-based strategies to promote youth well-being and combat the impact of suicide attempts.

## Introduction

Suicide remains a distressing and complex public health concern, notably affecting the pediatric population in the United States. Attempted suicide, involving intentional non-fatal self-harm, serves as a crucial indicator of mental health distress and a strong predictor of completed suicide [1-2]. Suicide ranks among the top nine leading causes of death for individuals aged 10 to 64 years. The National Vital Statistics System reported that in 2021, there were 48,183 reported deaths due to suicide, approximately one death every 11 minutes [3-5]. A report by the Substance Abuse and Mental Health Services Administration indicated that in 2021, about 12.3 million American adults considered suicide, with 3.5 million planning a suicide attempt and 1.7 million making attempts [4-5]. Notably, after death due to unintentional injuries, suicide is the second leading cause of death among 10- to 17-year-old adolescents in the United States [6].

In the United States, suicide is a leading cause of death among young students in grades 9-12. Its impact goes beyond those affected, causing emotional distress for families, friends, and communities [5]. Studying attempted suicide trends in the pediatric population is a crucial public health initiative to reduce its prevalence and devastating consequences. The Youth Risk Behavior Surveillance System (YRBSS), maintained by the Centers for Disease Control and Prevention (CDC), provides valuable data on health-related risk behaviors among high school students nationwide [7]. This database aids researchers and policymakers in developing evidence-based strategies to promote adolescent well-being.

This database offers distinct advantages for studying attempted suicide trends in the pediatric population. First, its comprehensive and nationally representative nature provides a broad perspective on prevalence across diverse demographics and time frames. This aids in understanding factors influencing attempted suicide rates and identifying vulnerable subpopulations for targeted interventions. Second, consistent updates allow for analyzing long-term trends, revealing shifts and patterns in suicidal behaviors. Detecting potential risks or protective factors informs targeted prevention efforts and mental health support systems. Third, it includes various health-related behaviors, enabling the exploration of associations between attempted suicide and other risk factors. Understanding these interconnected influences enhances comprehension of adolescent mental health and suicidal tendencies [6-8].

This study aims to analyze suicide attempts among the pediatric population; with suicide being a leading cause of death among young individuals, understanding attempted suicide trends is crucial for public health. The YRBSS database offers nationally representative data, enabling a comprehensive assessment of prevalence and risk factors across diverse groups. This study seeks to identify vulnerable subpopulations, explore long-term trends, and assess associations with risk behaviors. In addition to attempted suicide rates, this study will also explore various demographic and risk behavior variables that may be associated with suicide-related behaviors. Demographic variables, such as location, gender, race/ethnicity, and grade level, will be included in the analysis. Unraveling trends in adolescent attempted suicide rates is vital for researchers, policymakers, and mental health professionals. Through an in-depth analysis of these trends, we can gain valuable insights into the contributing factors of suicidal behaviors and develop targeted prevention and intervention strategies to address this urgent issue. The findings will inform evidence-based strategies, ultimately contributing to targeted prevention efforts and improved mental health support for youth in the United States.

## Materials and methods

Study population and timeframe

The study population consisted of students in grades 9-12. The surveys are administered every other year, representing the pediatric population most vulnerable to suicide-related behaviors. Data collection spanned the most recent available cycles of the YRBSS survey, ensuring an up-to-date analysis of attempted suicide trends in the United States [7]. Specifically, data from the past 16 survey cycles (1991-2021) were included to examine both short-term and potential long-term trends.

Data source

The primary data source for this study was the YRBSS database, maintained and periodically updated by the CDC. The YRBSS is a nationally representative survey collecting data on health-related risk behaviors among high school students in the United States. The survey, conducted biennially, covered various topics, including suicide-related behaviors, substance use, sexual behaviors, and mental health indicators. This dataset provided comprehensive information on attempted suicide rates among the pediatric population, serving as a valuable resource for the research [7]. Between 1991 and 2021, the YRBSS has gathered data from over five million high school students across more than 2,200 distinct surveys.

Variables and measures

The key variable of interest in this study was attempted suicide. The YRBSS survey collected data on self-reported suicide attempts in the past year using a standardized question: "During the past 12 months, how many times did you actually attempt suicide?" The respondents were provided with response options, ranging from "0 times" to "9 or more times" [7].

Data analysis

The data analysis was conducted in several stages using Microsoft Excel (version 2019, Microsoft Corporation, USA). First, descriptive statistics were computed to provide an overview of attempted suicide rates in the pediatric population across different demographic groups and survey cycles. This step allowed for the identification of any immediate trends or disparities in attempted suicide rates. In addition, time-trend analyses were conducted to investigate changes in attempted suicide rates over the past 16 survey cycles, enabling the identification of significant shifts or patterns in suicidal behaviors among adolescents during this period.

Ethical considerations

As this study utilized secondary data from the YRBSS database, ethical considerations primarily revolved around data confidentiality and anonymity. The CDC ensured strict data protection protocols, including de-identification of individual respondents, to safeguard their privacy. In addition, the research team complied with all relevant institutional and ethical guidelines while accessing and analyzing the data to ensure the responsible and ethical use of the dataset.

## Results

Attempted suicide by gender

**Figure 1:**
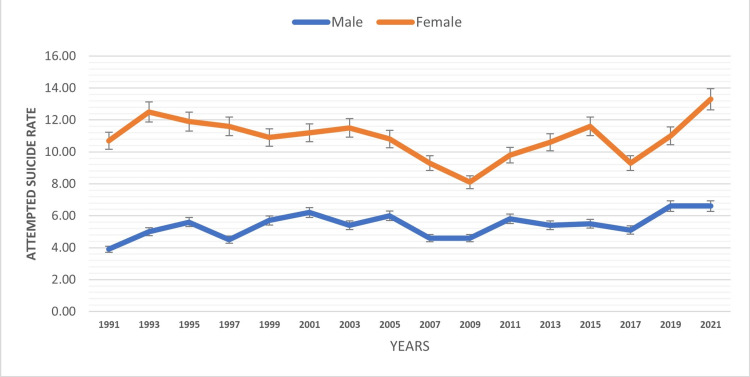
Attempted suicide rate based on gender

Gender-based analysis indicated significant differences in suicide attempts among the pediatric population (Table 1). Females reported a higher prevalence of attempted suicide with a rate of 13.3% (2021), while males exhibited a lower rate of 6.6% (2021). In addition, an increasing trend in attempted suicide rates were observed in both genders over the time period of 1991-2021 (Figure 1). These results emphasize the importance of gender-specific suicide prevention strategies to address the varying risk factors and support the mental well-being of both male and female adolescents.

**Table 1:** Attempted suicide rate based on gender classification (1991-2021) 95% CI: 95% confidence interval

Year	1991	1993	1995	1997	1999	2001	2003	2005	2007	2009	2011	2013	2015	2017	2019	2021
Total sample size	10,980	14,990	9,952	14,764	13,621	11,959	13,150	12,427	12,484	14,609	13,514	11,982	12,567	10,686	10,520	15,573
Total attempted suicide percentage (95% CI)	7.3 (6.4–8.3)	8.6 (7.9–9.5)	8.7 (7.9–9.5)	7.7 (6.8–8.7)	8.3 (7.3–9.4)	8.8 (8.0–9.7)	8.5 (7.4–9.6)	8.4 (7.6–9.3)	6.9 (6.3–7.6)	6.3 (5.7–7.0)	7.8 (7.1–8.5)	8.0 (7.2–8.9)	8.6 (7.6–9.6)	7.4 (6.5–8.4)	8.9 (7.9–10.0)	10.2 (9.4–11.0)
Female percentage (95% CI)	10.7 (9.4–12.1)	12.5 (11.2–13.9)	11.9 (10.4–13.6)	11.6 (9.7–13.7)	10.9 (9.2–12.9)	11.2 (10.2–12.3)	11.5 (10.1–13.0)	10.8 (9.8–12.0)	9.3 (8.2–10.4)	8.1 (7.2–9.0)	9.8 (8.9–10.7)	10.6 (9.4–11.9)	11.6 (9.7–13.7)	9.3 (7.7–11.1)	11.0 (9.7–12.5)	13.3 (12.0–14.7)
Male percentage (95% CI)	3.9 (3.2–4.9)	5.0 (4.2–5.8)	5.6 (4.5–7.0)	4.5 (3.7–5.4)	5.7 (4.6–7.1)	6.2 (5.2–7.5)	5.4 (4.4–6.6)	6.0 (4.9–7.4)	4.6 (4.0–5.2)	4.6 (3.9–5.5)	5.8 (5.0–6.7)	5.4 (4.5–6.3)	5.5 (4.7–6.4)	5.1 (4.3–6.1)	6.6 (5.5–8.1)	6.6 (5.8–7.5)

Attempted suicide based on territories and freely associated state’s location

The analysis of suicide attempts among the pediatric population based on territories and freely associated states revealed variations in attempted suicide rates (Table 2). Elevated rates were observed in some territories and freely associated states compared to the mainland United States. Palau reported a higher attempted suicide rate of 25.2% (2021), followed by the Northern Mariana Islands (17.6% in 2021) and Puerto Rico, Guam (9.9% in 2021). Meanwhile, the last updated survey data reported 19.4% (2013) in American Samoa, 16.5% (2019) in Guam, and 25.5% (2007) in Marshall Islands. In addition, no significant variations in attempted suicide rates were observed based on location over the time period of 1991-2021. These findings highlight the need for targeted mental health interventions in these regions to address the increased risk of suicide attempts among youth.

**Table 2:** Attempted suicide rate by territories and freely associated state’s location (1991–2021) 95% CI: 95% confidence interval

Year	1991	1993	1995	1997	1999	2001	2003	2005	2007	2009	2011	2013	2015	2017	2019	2021
American Samoa % (95% CI)	—	26.3 (22.8–30.0)	—	22.0 (19.2–25.0)	17.8 (15.0–20.9)	—	—	—	19.6 (18.3–21.0)	—	20.5 (19.0–22.1)	19.4 (17.9–20.9)	—	—	—	—
Guam % (95% CI)	—	—	20.5 (16.1–25.8)	19.5 (14.3–26.0)	—	23.5 (20.4–27.1)	—	—	16.9 (14.8–19.2)	—	17.0 (14.9–19.3)	18.6 (16.2–21.2)	16.9 (14.3–19.8)	20.6 (17.0–24.7)	16.5 (12.6–21.2)	—
Marshall Islands % (95% CI)	—	—	—	—	—	—	30.9 (28.5–33.4)	—	25.0 (22.7–27.5)	—	—	—	—	—	—	—
Northern Mariana Islands % (95% CI)	—	—	—	—	—	—	24.7 (22.8–26.7)	19.9 (18.4–21.5)	17.3 (15.9–18.9)	17.2 (15.8–18.7)	15.1 (13.7–16.6)	14.7 (13.4–16.2)	13.5 (12.2–14.9)	13.6 (12.2–15.3)	18.0 (16.6–19.6)	17.6 (16.1–19.2)
Palau % (95% CI)	—	—	—	—	18.0 (15.8–20.5)	23.4 (21.8–25.1)	—	—	25.3 (24.3–26.4)	20.1 (18.7–21.6)	17.9 (16.1–19.9)	26.9 (24.1–29.9)	18.0 (17.8–18.2)	—	—	25.2 (23.1–27.4)
Puerto Rico % (95% CI)	7.4 (6.2–8.7)	—	11.9 (10.7–13.3)	10.9 (9.8–12.1)	—	—	—	14.8 (12.7–17.2)	—	—	18.2 (15.7–21.0)	17.3 (14.1–21.1)	15.9 (14.2–17.8)	13.7 (11.0–17.0)	14.0 (11.7–16.6)	9.9 (8.0–12.0)

Attempted suicide by race/ethnicity

Examination of suicide attempts by race revealed distinct patterns (Table 3). As per the last survey's reported data from 2021, the American Indian/Alaska Native adolescents had the highest attempted suicide rate at 16.0%, followed by Black (14.5%), multiple race (11.7%), Hispanic (10.7%), Native Hawaiian or Other Pacific Islanders (9.7%), and White (9.0%) adolescents. Asian youth had the lowest attempted suicide rate at 6.4%. In addition, no significant variations in attempted suicide rates were observed based on racial and ethnic composition over the time period of 1991-2021. These findings underscore the significance of culturally tailored suicide prevention efforts to address the unique challenges faced by various racial and ethnic groups in different locations.

**Table 3:** Attempted suicide rate by race/ethnicity (1991-2021) 95% CI: 95% confidence interval

Year	1991	1993	1995	1997	1999	2001	2003	2005	2007	2009	2011	2013	2015	2017	2019	2021
American Indian or Alaska Native % (95% CI)	15.0 (4.1–42.2)	11.3 (5.9–20.5)	19.5 (9.7–35.2)	27.0 (17.3–39.6)	19.3 (10.4–33.0)	19.7 (11.9–30.8)	15.8 (9.2–25.8)	17.6 (12.1–24.9)	14.4 (8.8–22.8)	10.0 (5.5–17.4)	14.7 (12.1–17.9)	14.6 (7.9–25.5)	15.0 (8.9–24.1)	6.8 (2.2–19.3)	25.5 (12.6–44.6)	16.0 (10.5–23.7)
Asian % (95% CI)	12.5 (10.0–15.5)	9.3 (7.0–12.4)	7.6 (4.1–13.6)	6.9 (4.7–10.1)	7.0 (5.2–9.5)	10.0 (7.2–13.8)	14.0 (9.8–19.6)	6.9 (4.9–9.5)	5.6 (3.3–9.6)	4.0 (2.2–7.1)	10.8 (8.5–13.5)	9.5 (5.2–16.7)	7.8 (4.9–12.2)	5.7 (2.8–11.2)	7.7 (4.8–12.3)	6.4 (4.3–9.4)
Black % (95% CI)	6.6 (4.8–9.0)	8.4 (6.9–10.0)	9.5 (7.8–11.7)	7.3 (6.0–9.0)	7.3 (5.4–9.8)	8.8 (7.6–10.1)	8.4 (6.8–10.3)	7.6 (5.7–10.1)	7.7 (6.1–9.7)	7.9 (6.5–9.7)	8.3 (6.8–10.0)	8.8 (7.6–10.2)	8.9 (6.7–11.9)	9.8 (7.5–12.7)	11.8 (8.7–15.9)	14.5 (11.9–17.5)
Hispanic % (95% CI)	7.9 (6.2–9.9)	13.6 (12.1–15.1)	13.4 (10.4–17.3)	10.7 (8.6–13.3)	12.8 (10.9–15.1)	12.1 (10.5–13.9)	10.6 (9.3–12.1)	11.3 (9.8–12.9)	10.2 (9.0–11.6)	8.1 (7.1–9.3)	10.2 (8.8–11.8)	11.3 (9.7–13.1)	11.3 (9.9–13.0)	8.2 (6.7–10.1)	8.9 (7.1–11.1)	10.7 (9.5–12.0)
Native Hawaiian or Other Pacific Islander % (95% CI)	—	—	—	—	21.1 (11.7–35.1)	17.6 (9.3–30.8)	20.3 (9.6–37.8)	21.8 (11.0–38.5)	12.5 (6.3–23.3)	11.9 (8.0–17.3)	9.9 (5.1–18.1)	11.8 (5.7–22.8)	7.5 (2.3–21.6)	9.1 (4.4–18.0)	8.8 (2.4–27.2)	9.7 (4.2–20.8)
White % (95% CI)	6.7 (5.7–7.9)	7.7 (6.8–8.8)	7.6 (6.6–8.9)	6.3 (5.2–7.6)	6.7 (5.3–8.4)	7.9 (6.9–9.1)	6.9 (5.9–8.0)	7.3 (6.3–8.4)	5.6 (5.0–6.3)	5.0 (4.4–5.7)	6.2 (5.6–6.9)	6.3 (5.5–7.2)	6.8 (5.5–8.4)	6.1 (5.1–7.3)	7.9 (6.9–9.1)	9.0 (7.8–10.5)
Multiple race % (95% CI)	—	—	—	—	12.5 (9.8–15.9)	11.6 (8.0–16.5)	17.7 (11.2–26.8)	14.6 (10.5–20.0)	11.1 (7.6–15.9)	12.4 (8.7–17.4)	11.6 (8.3–16.0)	10.6 (7.8–14.3)	15.2 (11.5–19.8)	10.8 (8.2–14.2)	12.9 (9.7–17.1)	11.7 (9.1–14.9)

Attempted suicide by school grade level

**Figure 2:**
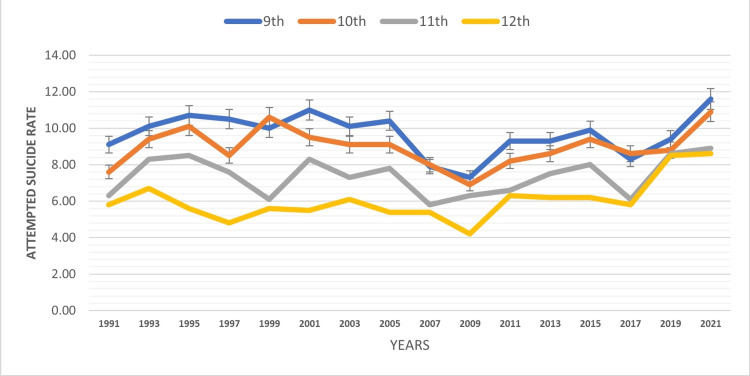
Attempted suicide rate by school grade level

The study examined suicide attempts among the pediatric population across different school grade levels (Table 4). As per the last survey's reported data from 2021, the results indicated a reverse pattern, with ninth-grade students reporting higher attempted suicide rates at 11.6%. Tenth-grade students exhibited the rate at 10.9%, followed by 11th- (8.9%) and 12th-grade students (8.6%). In addition, an increasing trend in attempted suicide rates were observed across all classes over the time period of 1991-2021 (Figure 2). This counterintuitive finding suggests that suicide prevention efforts should not solely target older adolescents but also focus on supporting younger students to mitigate the risk of suicidal behaviors.

**Table 4:** Suicide attempts rate by school grade level (1991-2021) 95% CI: 95% confidence interval

Year	1991	1993	1995	1997	1999	2001	2003	2005	2007	2009	2011	2013	2015	2017	2019	2021
9th % (95% CI)	9.1 (7.2–11.4)	10.1 (8.6–11.9)	10.7 (8.9–12.7)	10.5 (8.4–13.0)	10.0 (8.4–11.9)	11.0 (9.0–13.2)	10.1 (8.7–11.6)	10.4 (8.8–12.3)	7.9 (6.8–9.1)	7.3 (6.2–8.5)	9.3 (8.0–10.8)	9.3 (8.2–10.4)	9.9 (8.5–11.5)	8.3 (7.3–9.4)	9.4 (7.9–11.1)	11.6 (10.3–13.0)
10th % (95% CI)	7.6 (6.0–9.6)	9.4 (8.1–10.9)	10.1 (8.3–12.2)	8.5 (7.1–10.1)	10.6 (8.6–13.0)	9.5 (8.0–11.3)	9.1 (7.8–10.7)	9.1 (7.9–10.6)	8.0 (6.9–9.4)	6.9 (5.8–8.2)	8.2 (7.5–9.1)	8.6 (6.9–10.8)	9.4 (7.6–11.6)	8.6 (6.8–10.8)	8.8 (7.4–10.5)	10.9 (9.5–12.4)
11th % (95% CI)	6.3 (4.6–8.5)	8.3 (6.8–10.0)	8.5 (6.5–11.1)	7.6 (6.1–9.3)	6.1 (4.8–7.7)	8.3 (7.0–9.8)	7.3 (6.0–8.9)	7.8 (6.3–9.5)	5.8 (4.8–6.9)	6.3 (5.3–7.3)	6.6 (5.5–7.9)	7.5 (6.2–9.2)	8.0 (6.8–9.5)	6.1 (4.9–7.7)	8.6 (7.1–10.4)	8.9 (7.5–10.5)
12th % (95% CI)	5.8 (4.7–7.3)	6.7 (5.3–8.6)	5.6 (3.9–8.1)	4.8 (3.6–6.3)	5.6 (4.4–7.0)	5.5 (4.6–6.6)	6.1 (4.8–7.6)	5.4 (4.3–6.8)	5.4 (4.4–6.5)	4.2 (3.4–5.2)	6.3 (5.4–7.4)	6.2 (4.9–7.8)	6.2 (4.9–7.9)	5.8 (4.5–7.6)	8.5 (6.8–10.6)	8.6 (7.7–9.6)

Based on sexual orientation

Attempted Suicide by Sexual Orientation

The study examined suicide attempts among the pediatric population based on sexual orientation. The results revealed significant disparities in attempted suicide rates among different sexual orientation groups. As per the last survey's reported data from 2021, bisexual (25.4%); lesbian, gay, and bisexual (LGB; 24.1%); and gay or lesbian (19.1%) adolescents reported considerably higher attempted suicide rates compared to their heterosexual counterparts (6.3%). These findings underscore the urgent need for tailored and inclusive suicide prevention efforts to address the mental health challenges faced by LGB, gay or lesbian, and bisexual youth.

Attempted Suicide Based on Different Types of Sexual Contact** **

The study examined suicide attempts among the pediatric population based on sexual contact. The results revealed significant differences in attempted suicide rates among adolescents who reported different types of sexual contact (Table 5). As per the last survey's reported data from 2021, adolescents who reported both-sex (36.7%) and same-sex-only sexual contact or both (32.9%) had notably higher attempted suicide rates compared to those who reported opposite-sex sexual contact (11.9%) or no sexual contact (4.6%). In addition, an increasing trend in attempted suicide rates were observed across all types of sexual contacts over the time period of 2015-2021. These findings emphasize the importance of considering sexual orientation in suicide prevention efforts and addressing the unique mental health challenges faced by adolescents with same-sex sexual contact. 

**Table 5:** Suicide attempts rate by sexual contact (2015-2021) 95% CI: 95% confidence interval

Year	2015	2017	2019	2021
Opposite sex only % (95% CI)	9.7 (8.6–10.9)	8.1 (7.0–9.5)	9.3 (7.9–10.8)	11.9 (10.8–13.1)
Same sex only % (95% CI)	20.6 (12.9–31.3)	17.4 (13.0–22.9)	25.5 (17.7–35.3)	22.6 (17.9–28.2)
Both sexes % (95% CI)	29.9 (25.5–34.7)	25.6 (20.3–31.7)	32.3 (27.4–37.8)	36.7 (31.1–42.7)
Same sex only or both sexes % (95% CI)	27.6 (23.5–32.1)	23.8 (19.5–28.6)	30.3 (25.9–35.0)	32.9 (28.9–37.2)
No sexual contact % (95% CI)	4.2 (3.3–5.3)	4.2 (3.5–5.2)	4.8 (4.0–5.8)	4.6 (4.0–5.2)

Overall, these results provide crucial insights into suicide attempts among the pediatric population in the United States based on territories and freely associated state’s location, gender, race/ethnicity, school grade level, and sex orientation. Tailoring prevention and intervention strategies to address these disparities is vital for effectively combating the alarming rates of suicide attempts among adolescents and promoting the mental well-being of youth across diverse geographic and demographic groups.

## Discussion

The present study utilized data from the YRBSS to comprehensively analyze suicide attempts among the pediatric population in the United States. The complex interplay of factors has been explained in detail with the tabular presentation in the Results section. The findings have significant implications for tailored suicide prevention efforts and mental health support systems to effectively address the alarming rates of suicide attempts among youth.

The gender-based analyses demonstrated significant disparities in suicide attempts among the pediatric population. Females exhibited higher attempted suicide rates compared to males. These findings underscore the vulnerability of certain subgroups within the pediatric population and emphasize the important findings for specific prevention program. Addressing stigma, discrimination, and providing targeted mental health support for vulnerable groups is critical steps to reduce suicide risk. These gender-specific results were consistent with previously published studies [9-12]. However, these findings were not aligned with a study conducted by Bridge et al., which reported a higher suicide rate among elementary school boys compared to girls in the United States [13].

The study revealed variations in attempted suicide rates based on territories and freely associated states' locations. Certain regions, such as Palau, American Samoa, Guam, Marshall Islands, and the Northern Mariana Islands, exhibited higher rates. These findings indicate the need for targeted mental health interventions in these areas to address the increased risk of suicide attempts among adolescents. Factors, such as access to mental health services, cultural norms, and socioeconomic disparities, may play a role in shaping regional suicide rates and warrant further investigation. More research is needed to enhance the comparability of attempted suicide rates based on territories and freely associated states' locations.

The study also highlighted racial and ethnic disparities in attempted suicide rates among adolescents. American Indian/Alaska Native adolescents had the highest attempted suicide rate, followed by Black and Hispanic youth, while other races, such as Native Hawaiian or Other Pacific Islander, White, and Asian youth, had the lowest attempted suicide rate. These findings underscore the significance of culturally tailored suicide prevention efforts that consider the unique challenges faced by various racial and ethnic groups. A consistent finding was reported in previously conducted studies, showing that American Indian/Alaska Native adolescents had the highest attempted suicide rate [9,14-16].

Surprisingly, the study revealed a reverse pattern in attempted suicide rates based on school grade level, with younger students reporting higher rates. Ninth-grade students exhibited the highest attempted suicide rate, followed by decreasing trends among the 10th-, 11th-, and 12th-grade students. This counterintuitive finding highlights the importance of early intervention and mental health support for younger adolescents. Comprehensive suicide prevention efforts should not solely focus on older students but also address the unique challenges faced by younger students to mitigate the risk of suicidal behaviors. This finding is consistent with previous research conducted by Stephenson et al. among high school students [9].

The results of this study revealed significant disparities in suicide attempts based on sexual orientation. LGB adolescents exhibited considerably higher attempted suicide rates compared to their heterosexual peers. These findings were further strengthened by previously conducted studies with similar results [9-10,17-21]. The study findings underscore the importance of initiating mental support programs.

The findings of this study may be subject to certain limitations. Because the data are self-reported, there might be recall bias or social desirability bias in responses related to sensitive topics, such as suicide attempts. To address this limitation, future studies could consider incorporating additional validation measures, such as cross-referencing self-reported data with external sources or utilizing more confidential and anonymous survey methods, to enhance the accuracy and reliability of responses, particularly concerning sensitive topics, such as suicide attempts. Moreover, the YRBSS survey captures attempted suicide rates among high school students, which may not be representative of all adolescents in the pediatric population. The study is also limited to analyzing associations rather than establishing causation due to its cross-sectional nature. In addition, the dataset does not capture all potential confounding factors, such as family dynamics, access to firearms, or mental health history. Despite these limitations, the use of a nationally representative dataset, such as the YRBSS, allows for valuable insights into attempted suicide trends among the pediatric population, aiding in the formulation of evidence-based preventive measures and intervention strategies to address this critical public health concern. Future research should explore these factors to gain a more comprehensive understanding of suicidal behaviors among adolescents.

## Conclusions

The study reveals significant disparities in suicide attempts among the pediatric population in the United States. Females had higher attempted suicide rates than males, and rates increased over time for both genders. Certain territories and freely associated states reported elevated rates compared to mainland United States. American Indian/Alaska Native adolescents had the highest attempted suicide rate by race. Bisexual, LGB, and gay or lesbian adolescents had considerably higher rates based on sexual orientation. The findings of this study, which indicate higher suicide rates among females compared to males, as well as among ninth graders compared to 12th graders, and varying rates based on sexual orientation, will contribute to advancing research in this field and guide the development of prevention programs for youth mental health and suicide prevention. Tailored interventions based on regional, gender, racial/ethnic, sexual orientation, and grade level disparities are essential to promote the mental well-being of the pediatric population. Implementing comprehensive suicide prevention strategies and fostering supportive environments will be crucial in reducing the alarming rates of suicide attempts among youth and creating a safer and healthier future for the next generation.
